# Relationships between Willingness to Participate in the Nursing Clinical Ladder Program and Its Related Factors among Clinical Nurses

**DOI:** 10.3390/healthcare10020369

**Published:** 2022-02-14

**Authors:** Yi-Hui Li, Man-Chun Chou, Ling-Dai Lin, Ching-Ching Tsai, Mei-Hsiang Lin

**Affiliations:** 1 Department of Nursing, Kaohsiung Chang Gung Memorial Hospital, Kaohsiung 83301, Taiwan; 97921huil@adm.cgmh.org.tw (Y.-H.L.); anny5626@adm.cgmh.org.tw (M.-C.C.); 0829.lld@yahoo.com.tw (L.-D.L.); dily61@yahoo.com.tw (C.-C.T.); 2 School of Nursing, Chung Hwa University of Medical Technology, Tainan 717302, Taiwan; 3 School of Nursing, Fooyin University, Kaohsiung 83102, Taiwan; 4 School of Nursing, National Taipei University of Nursing and Health Sciences, Taipei 112303, Taiwan

**Keywords:** clinical ladder systems, motivation, level of satisfaction, professional competence

## Abstract

The professional nursing competence ladder system can effectively inspire nurses’ work morale, improve quality of life, and avoid the issue of senior staff leaving the clinical setting. The aim of this study was to explore the willingness to participate in the professional nursing competence ladder system and its related factors among nurses. A cross-sectional study design with a structured questionnaire was used. Purposive sampling was employed, and 696 nurses who qualified to be promoted as N2 were recruited from a medical center in southern Taiwan. The results showed most nurses were willing to participate in the nursing ladder system. There were significant differences between willingness to participate in the ladder system and age, education level, as well as promotion experience. This study emphasizes the importance of intensifying internal encouraging factors and strengthening external encouraging factors to improve participation rates. Healthcare institutions could provide instruction on case report writing to increase nurses’ willingness to participate in the clinical ladder program.

## 1. Introduction

The clinical ladder program, which was developed in 1970 [1], is a grading structure that defines different levels of clinical practice for nurses. The clinical ladder program recognizes and rewards nurses’ contributions to quality care and highlights evidence- based practices that positively influence patient outcomes [2,3]. Additionally, the clinical nursing ladder system refers to a hierarchical structure that is associated with an individual’s clinical abilities and proficiency growth. A four-level ladder system, which encompasses N1 (responsible for basic nursing), N2 (critical care nursing), N3 (in charge of education and holistic nursing), and N4 (responsible for research and specialized nursing), has been widely adopted by medical centers for more than 20 years in Taiwan [4]. Studies showed that the nursing clinical ladder program facilitated the gradual development, increases self-affirmation, alters working attitudes, and improves the morale of nurses [5,6,7,8]. One study investigated N2 nurses who participated in the clinical ladder program and found that participation improved their clinical, administrative, and teaching abilities [9]. Moreover, some studies pointed out that such programs lead to positive outcomes for nurses through increased nurse satisfaction and better retention and recruitment [10,11]. These findings indicate that participation in the clinical ladder program improves professional competence and further enhances the quality and safety of nursing services. Notably, the qualifications of promotion under the clinical ladder program for primary care nurses in Taiwan were revised in 2004. Nurses wishing to be promoted from level N2 to N3 should demonstrate competence in clinical practices, conducting research, teaching, and administrative tasks. In order to build up their ability to conduct research, a nurse should have completed 20 h of on-service training and participated in academic activities for 12 hours. In addition, they must pass the Taiwan Nurses Association (TNA) case report writing [12]. Pai et al. [13] investigated 314 nursing professionals and found that 86.6% of them were willing to participate in the ladder program. Moreover, those willing to participate in the program demonstrated significantly better “self-discipline and professional development” of nursing competence than those not willing to participate. Bjørk et al. [14] found that reasons for nurse participation in the ladder program include the opportunity to refresh nursing knowledge and skills (66.5%) and self-development (61%). Another study found that those who were willing to participate in the program had significantly higher self-perceived nursing competence than those who were not [4]. Different curriculum regulations reflect differences in educational philosophy. In Australia, undergraduate nursing courses include basic nursing, nursing research and application, biological sciences, social sciences, and clinical practice [15]. In Taiwan, most nursing curricula follow the traditional discipline-centered model, mainly including basic medical courses, nursing, and clinical practice for nursing school. Such curricula emphasize the integrity of theoretical knowledge systems and disciplines [12]. At this point, we speculated that the clinical ladder system can provide a way of encouraging and continuing education for updating one’s professional skills.

One study showed that incentives leading to increased employee motivation are important. For this reason, the creation of adequate motivation systems and the application of an appropriate program of incentives are important for providing better efficiency and quality of healthcare services [16]. Factors influencing nursing staff to participate in the clinical ladder system include motivation, satisfaction, sense of achievement, and stress [9]. Motivation refers to an inner process that drives one to take action under the influence of an external factor. This process is dynamic and continuous and results in practice or action [17]. Bjørk et al. [14] highlighted that 46.2% of nurses would be willing to participate in the ladder program if a promotion increased their salary. Juang [9] suggested that “rewards”, “days off offered by superiors”, and “superiors actively implementing the ladder system” are significant motivators for nurses to participate in the clinical ladder program. However, another influence on participation in the ladder program is the pass rate of the N3 case report writing requirement. One study revealed that 90% of nurses believed it was relatively difficult to pass the N3 case report writing requirement [18]. In addition, previous studies found that it generally takes 7.07 ± 5.34 months for a nurse to complete a case report. Writing a case report may be more difficult than taking care of patients for nurses and may bring additional mental stress and affect their quality of life. As a result, their willingness and motivation to participate in promotion assessment via case report writing is relatively low [4,18]. Satisfaction is the level of joyfulness or disappointment of a perception or expectation toward a specific thing [19]. Many different factors have been proposed as precursors of job satisfaction. Most of these factors under study relate to aspects of the work situation [20]. Juang [9] indicated that nurses were satisfied with “superiors offering more official leaves” and “official leaves offered for participating in the program” and were less satisfied with a “promotion bonus based on the competence levels” and “course arrangement”. However, the job responsibilities and salary adjustment after promotion used in the study were not satisfactory.

Nursing competence refers to the broad personal abilities developed through professional nursing training and is considered the outcome of nursing education [21]. Some studies suggested that nurses recognize the effectiveness of the clinical ladder program in enhancing professional competence [1,9]. Other studies highlighted that when job levels were divided based on personal ability, nurses’ clinical experience and abilities were not only recognized, giving them a sense of belonging, but on a personal level, nurses also obtained a sense of achievement [9,22]. Juang [9] found that nurses felt a sense of achievement when participating in the clinical ladder program, and that advancing their professional competence and receiving approval and respect were particularly important. Stress derived from the promotion process is worth noting. Stress is a subjective perception derived from one’s explanation of his/her relationship with the environment. Several studies found that factors such as age, seniority, and marital status are related to willingness to participate in the clinical nursing ladder program [4,9,23,24]. Additionally, Hsu et al. [5] suggested that nurses with higher seniority show better competence. However, Juang [9] found that motivation to participate was not affected by age.

The aforementioned literature reveals the significance of the clinical ladder system among clinical nurses. However, Li and Zhou [25] studied 701 N2 nurses qualified to be promoted to level N3 in a medical center located in the south of Taiwan and found only 3.70% (*n* = 26) of the nurses were willing to participate in a promotion assessment. This finding showed that the participation of N2 nurses in a promotion assessment under the clinical ladder program is low. Given the ever-changing medical technologies and the fact that professional knowledge and skills are only current for approximately 2 to 5 years [9], nurses without career progression cannot obtain advanced knowledge and skills, which influences quality of care. This study aimed to understand nurses’ willingness to participate in the nursing clinical ladder program, and any influencing factors. Hopefully, the results can serve as a reference for nursing administrators when educating nursing professionals on participating in the clinical ladder program, deliver adequate human resource management, and lead to improved nursing care and performance quality.

## 2. Materials and Methods

### 2.1. Design and Sample

This study employed a cross-sectional design. The purposive sampling method was adopted to recruit N2 nurses who were qualified for promotion assessment from a medical center located in the south of Taiwan. A structured questionnaire comprised the study tool; 736 questionnaires were distributed and 696 were returned, yielding a return rate of 94.5%.

### 2.2. Measurements

We obtained participant demographic data including age, seniority, seniority of current position, education level, marital status, number of children, experience with report writing, experience of promotion application, and department of service.

#### 2.2.1. Questionnaire Related to Participation in the Nursing Clinical Ladder Program

Based on previous literature, a willingness to participate in the nursing clinical ladder program scale that comprises 65 items was developed to measure motivation, satisfaction, sense of achievement, stress, and advancement of professional competence [9]. The original questionnaire was revised based on the opinions of six scholars and experts, and the Cronbach’s α value was 0.83. This study’s questionnaire was designed based on the research tool developed in Juang’s [9] study, which examined willingness to participate in the professional nursing competence ladder system. In this study, the content validity of the revised questionnaire was reviewed by three expert scholars and contained 50 items.

The Content Validity Index(CVI) for the revised overall questionnaire, motivation, level of satisfaction, professional competence, sense of achievement, and stress was 0.98, 0.93, 0.94, 0.98, 0.97, and 0.86, respectively. The Cronbach’s α for the overall questionnaire, motivation, level of satisfaction, professional competence, sense of achievement, and stress was 0.91, 0.87, 0.86, 0.97, 0.92, and 0.87, respectively. A five-point Likert scale was applied, where a higher score indicates stronger motivation, satisfaction, recognition of the effectiveness in improving professional competence, sense of achievement, and stress regarding participation in the clinical ladder program. 

#### 2.2.2. Willingness to Participate in the Clinical Ladder Program

Participants were divided into those who were willing to participate and those who were not.

### 2.3. Data Analysis

Data were analyzed using SPSS/PC software version 20.0 (IBM Corp, Armonk, NY, USA). Descriptive statistical methods were used to analyze both the characteristics of the study participants according to demographics and the nursing competence and related factors of nurses participating in a clinical ladder system. The distributions of participants’ demographic data and data relating to the clinical ladder system compared to their promotion intention were calculated using a chi-square test and *t*-test. Finally, logistic regression analysis was conducted to identify factors associated with the willingness to participate in clinical ladder systems among participants. The statistical significance was when *p* < 0.05.

### 2.4. Ethical Considerations

The study protocol and consent form were approved by the institutional review board of Chang Gung Memorial Hospital in Taiwan (Approval Code: 98-1887B). We used items that maintained participants’ anonymity in this research so as to not identify individuals based on their responses. Additionally, we stressed to the participants that any information provided in the questionnaires was for academic research only. In addition, the participants were informed that the research process would not involve any risk or comorbidity, and that they had the right to withdraw from the study without penalty at any time.

## 3. Results

### 3.1. Demographics

The study sample comprised 696 participants, and 68.7% of them were willing to participate in the clinical ladder program. The nurses’ mean age, seniority, and seniority of the current position of those willing to participate was 28.88 ± 3.47, 6.56 ± 3.40, and 5.02 ± 3.44 years, respectively. Approximately 31.3% (*n* = 218) of the study participants were unwilling to participate in the clinical ladder program, and their mean age, seniority, and seniority of their current position was 29.50 ± 3.56, 7.05 ± 3.60, and 5.42 ± 3.59 years, respectively (Table 1).

### 3.2. Distribution and Variance of Influences on Participation in the Clinical Ladder Program

According to the results from nurses who were willing to participate in the program, “days off offered by superiors” ranked at the top of the motivation section. Regarding the satisfaction section, “the more days off from work offered for participating in the program, the more satisfied I am” had the highest score. Regarding the section on advancement of professional competence, “enhancing writing capacity” had the highest score. In the section on sense of achievement, “receiving affirmation” ranked at the top. “My case report requirement made me feel very stressed” had the highest score in the stress section. Table 2 shows the distribution of nursing competence and influencing factors among nurses who were willing and unwilling to participate in the clinical ladder program.

Notably, between both groups of nurses, those who were willing to participate in the assessment showed the highest average score in the section on sense of achievement (3.63 ± 0.63) and the lowest average score in the satisfaction section (3.28 ± 0.59). In contrast, those who were unwilling to participate in the program had the highest average score in the stress section (3.26 ± 0.57) and had the lowest average score in the satisfaction section (2.80 ± 0.60). Furthermore, significant differences were found in terms of motivation, satisfaction, professional competence, and sense of achievement between the two groups (t = −9.97; t = −10.03; t = −8.71; and t = −10.62, *p* < 0.01) (Table 3).

### 3.3. Influencing Factors Regarding Participation in the Clinical Ladder Program

Using logistic regression analysis to predict the factors influencing nurses’ participation in the clinical ladder program, their motivation, satisfaction, and sense of achievement were revealed to be significant variables. Compared to nurses who were unwilling to participate in the program, those who were willing to participate showed a 1.08 (95% CI: 1.03–1.13) times greater motivation, a 1.10 (95% CI: 1.01–1.19) times higher satisfaction, and a 1.14 (95% CI: 1.06–1.23) times stronger sense of achievement. In other words, those who were willing to participate in the program demonstrated better motivation, satisfaction, and sense of achievement than those who were not.

## 4. Discussion

The study sample comprised 696 nurses, and 68.7% (*n* = 478) of them intended to participate in the clinical ladder program. Compared to the willingness rate of 86.6% found in the study by Hsu et al. [5], participants in this study expressed lower participation willingness. Chen [6] highlighted that nurses’ low participation willingness could be related to the 40% pass rate of the case report writing requirement. According to the results of this study, the items with low scores from the group of nurses who were willing to participate in the program were “low pass rate of the case report writing”, “the requirement to write case reports”, and “superiors actively implementing the ladder system”, which were the factors reducing participation willingness. However, Juang [9] had a different finding regarding the influence of “superiors actively implementing the ladder system”. In addition, in this study, we found that age had an influence on participation. However, this finding is different from the findings of a study led by Juang [9]. In Juang’s study, there were no significant differences between age and willingness to participate in ladder system training. In this study, experience of promotion was found to be related to participation willingness, possibly because case report writing is required for promotion. However, the pass rate of the case report writing requirement is low, and it is a time-consuming process, taking 7.07 ± 5.34 months on average [4,18]. Our results revealed that nurses from the departments of emergency care and intensive care showed higher participation willingness, followed by those from the departments of internal medicine, surgery, and gynecology and pediatrics. Nurses working in operation rooms expressed the lowest participation willingness. According to a previous study, which investigated nurses’ attitudes toward the clinical ladder system, nurses from the department of obstetrics and gynecology expressed the highest interest in the program, followed by those from the departments of pediatrics, surgery, and internal medicine [26]. These inconsistent findings could be attributed to attitudes toward program implementation among different supervisors.

The results of this study showed that education level was significantly related to participation willingness, although it was not significant in the logistic regression model. Pai et al. [13] and Hsu et al. [5] found that nurses demonstrating better nursing abilities were more willing to participate in the ladder program. Wu et al. [27] studied the influence of self-evaluated professional development on participation in the clinical ladder program and found that nurses with a higher education level had better self-evaluated professional development. However, the relationships among self-evaluated professional competence, education level, and participation willingness were not examined in this study. This study discovered that motivation was a significant factor that influenced participation willingness; nurses with a higher score in the motivation section showed higher willingness to participate in the clinical ladder program. This study also found that “official leaves offered for participating in the program” and “superiors actively implementing the ladder system” were the best motivators. This finding is consistent with that in Juang’s [9] study, though it differs from that of Bjørk et al. [14], where salary was found to reinforce motivation.

The result of this study further identified that those with a higher satisfaction toward the clinical ladder program were more willing to participate in the program. This finding is consistent with that reported by Juang [9]. In addition, nurses with a higher score in the sense of achievement section were more willing to participate in the program, which is consistent with the finding of Shin et al. [22]. Regarding stress derived from the promotion assessment, the case report writing item had the highest score, suggesting that this was the primary source of stress affecting nurses’ willingness to participate. Nurses must pass the case report writing to obtain a promotion within the clinical ladder [12]. However, some difficulties were also encountered in applying the clinical ladder and ensuring time for additional tasks such as providing support and conducting assessment meetings during business hours. In addition, nurses’ working hours remained a problem in this study. Studies have shown that writing reports is difficult, and the progression process is time-consuming [28,29], resulting in a relatively low willingness and motivation to participate in promotions. Our finding of problematic working hours is consistent with that of Wang et al. [30], who examined the job stress and social support of 562 nursing staff in Taiwan. This study was conducted in a single medical center, which reduces its external validity and transferability. Moreover, interviews, as opposed to questionnaires, not only facilitate the collection of more objective and accurate data to verify study results and identify influences on participation in the clinical ladder program, but they also improve the representativeness of the study sample. A qualitative study using interviews is recommended for future studies to identify areas for improvement regarding the application of the clinical ladder system.

Some limitations of this study should be noted. First, the items assigned to scales both in the original study and the present study were based on face validity. The factor analysis of the nursing clinical ladder program questionnaires was not examined at this time. Future studies should perform exploratory and confirmatory factor analysis to determine the conformity of the scales’ factor structure with the theoretical model. Second, since the purposive sampling method was employed in this study, the geographical limitation of the sample restricts the extent of generalization. Future studies could adopt a longitudinal design to confirm the nurses’ willingness to participate in the clinical ladder program.

## 5. Conclusions

Based on the results of this study, information was obtained regarding the practical application of the nursing clinical ladder in a medical hospital in Taiwan. This study discovered that case report writing is the primary source of stress associated with the clinical ladder program. Therefore, in addition to intensifying internal encouraging factors, offering official leaves for participating in the program, providing salary adjustments, offering promotion opportunities, and strengthening external encouraging factors to improve participation rates, healthcare institutions could provide instruction on case report writing to increase nurses’ willingness to participate in the clinical ladder program.

## Figures and Tables

**Table 1:** Characteristics of the participants (*N* = 696).

Variable	Unwilling to Participate		Willing to Participate		t/χ^2^	*p*
	*n* = 218 (31.3%)		*n* = 478 (68.7%)			
	*n* (%)	Mean (SD)	*n* (%)	Mean (SD)		
Age		29.50 (3.56)		28.88 (3.47)	2.16	0.03
Years of nursing experience		7.05 (3.60)		6.56 (3.40)	1.71	0.08
Years of job position experience		5.42 (3.59)		5.02 (3.44)	1.40	0.16
Education level						
College (inclusive)	82 (37.6)		134 (28.0)		6.42	0.01
University/institute of technology (or more)	136 (64.1)		344 (72.0)			
Marital status						
Married	73 (33.5)		132 (27.6)		2.48	0.11
Unmarried	145 (66.5)		346 (72.4)			
Children status						
No	158 (72.5)		369 (77.2)		1.81	0.17
Yes	60 (27.5)		109 (22.8)			
Case report experience						
No	100 (45.9)		234 (49.0)		0.57	0.45
Yes	118 (54.1)		244 (51.0)			
Project/research experience						
No	204 (93.6)		450 (94.1)		0.08	0.77
Yes	14 (6.4)		28 (5.9)			
Promotion experience						
No	139 (63.8)		263 (55.0)		4.68	0.03
Yes	79 (36.2)		215 (45.0)			
Service departments						
Medical department	57 (26.1)		141 (29.5)		7.35	0.11
Surgery	44 (20.2)		89 (18.6)			
Women pediatrics	40 (18.3)		64 (13.4)			
Operating room	17 (7.8)		23 (4.8)			
Emergency/ICU	60 (27.5)		161 (33.7)			

**Table 2:** Nursing competence and influencing factors.

Variable	Willing to Participate (*n* = 478)			Unwilling to Participate (*n* = 218)		
	Mean	SD	Rank	Mean	SD	Rank
**Motivation**						
Official leaves offered by superiors	4.08	0.69	1	3.57	0.81	1
Salary adjustment	3.93	0.82	2	2.82	0.87	7
Promotion of job position	3.83	0.76	3	2.55	0.96	9
Support from peers	3.54	0.81	4	3.45	0.98	2
Encouragement from families	3.53	0.90	5	3.18	0.92	3
Encouragement from superiors	3.49	0.82	6	2.93	0.86	4
Superiors actively implementing the ladder system	3.43	0.85	7	2.93	0.85	5
Lack of motivation due to writing case reports *	3.01	0.97	8	2.83	0.82	6
Lack of motivation due to low passing rate of case reports *	2.68	1.05	9	2.79	0.77	8
**Level of satisfaction**						
The more days off provided by superiors to participate in the ladder system training, the more satisfied I am	3.58	0.91	1	2.62	0.76	6
Arrangement of the ladder system course	3.40	0.73	2	2.74	0.82	4
Satisfied with consideration for promotion and advancement	3.33	0.69	3	2.88	0.86	3
Feel satisfaction with current nursing tasks	3.30	0.84	4	2.94	0.71	1
Feel satisfaction with the current nursing ladder system	3.08	0.84	5	2.89	0.65	2
Discrepancy in salaries	3.05	0.82	6	2.72	0.76	5
**Professional competence**						
Enhance writing ability	3.66	0.74	1	3.17	0.77	3
Enhance teaching ability in clinic	3.65	0.72	2	3.23	0.74	1
Enhance ability to conduct nursing assessments	3.64	0.77	3	3.17	0.77	4
Enhance ability to evaluate oneself and others	3.64	0.74	4	3.15	0.77	6
Enhance patient education ability	3.63	0.75	5	3.13	0.74	7
Enhance ability to implement care plans	3.62	0.73	6	3.17	0.79	5
Enhance ability to complete nursing reports	3.60	0.75	7	3.07	0.86	11
Enhance problem analysis and solving ability	3.59	0.77	8	3.11	0.77	9
Improve quality care ability	3.57	0.73	9	3.19	0.76	2
Enhance ability to plan nursing career	3.56	0.81	10	3.01	0.77	14
Improve communication skills	3.55	0.79	11	3.12	0.78	8
Increase ability to deal with crises	3.54	0.76	12	3.09	0.75	10
Increase ward management ability	3.44	0.77	13	3.04	0.76	12
Enhance knowledge related to medical law	3.42	0.80	14	3.03	0.82	13
**Sense of achievement**						
Receiving affirmation	3.76	0.69	1	3.15	0.83	2
Being respected	3.72	0.72	2	3.21	0.77	1
Sense of achievement due to improved nursing ability	3.68	0.70	3	3.12	0.76	3
I feel honored for receiving a higher grade on my nursing report	3.64	0.77	4	3.05	0.81	4
Commitment to nursing tasks due to sense of achievement	3.38	0.80	5	2.81	0.82	5
**Stress**						
My case report requirement made me feel very stressed	3.69	0.86	1	3.57	0.88	2
Feel anxious	3.65	0.85	2	3.44	0.93	4
Superior actively implementing the system	3.48	0.94	3	3.32	0.93	7
Using a pencil during the examination made me feel very stressed	3.48	0.91	4	3.18	0.93	9
Feel nervous and panicky	3.44	0.91	5	3.15	0.92	10
Having seniority with a lower position made me feel very stressed	3.29	0.92	6	3.29	0.96	8
Trouble sleeping	3.28	1.00	7	2.96	0.93	12
Unwilling to participate in the ladder system due to a lack of confidence	3.24	0.91	8	3.37	1.00	6
Easily angered and flighty	3.09	0.99	9	2.91	0.92	13
Difficulty dealing with daily chores	3.08	0.98	10	3.56	0.86	3
Made me feel unhappy	3.06	1.01	11	3.02	0.91	11
Suffer chronic pain such as headaches and abdominal pain	2.84	1.01	12	3.67	0.91	1
I cannot work	2.83	0.94	13	3.40	0.92	5
Support from family can reduce my stress *	2.37	0.76	14	2.83	0.77	16
Support from peers can reduce my stress *	2.34	0.74	15	2.88	0.70	14
Support from superiors can reduce my stress *	2.34	0.71	16	2.87	0.75	15

* Reverse scoring questions.

**Table 3:** Distribution and variance of influences on participation in the clinical ladder program between the two groups of nurses (*N* = 696).

Variable Promotion Intention	Unwilling to Participate *n* = 218	Willing to Participate *n* = 478	t	*p*
	Mean (SD)	Mean (SD)		
Motivation	3.14 (0.54)	3.57 (0.50)	−9.97	0.00
Level of satisfaction	2.80 (0.60)	3.28 (0.59)	−10.03	0.00
Professional competence	3.12 (0.66)	3.58 (0.63)	−8.71	0.00
Sense of achievement	3.06 (0.71)	3.63 (0.63)	−10.62	0.00
Stress	3.26 (0.57)	3.33 (0.61)	−1.42	0.15

## Data Availability

All relevant datasets in this study are described in the manuscript.
